# Light‐Controllable Digital Coding Metasurfaces

**DOI:** 10.1002/advs.201801028

**Published:** 2018-08-01

**Authors:** Xin Ge Zhang, Wen Xuan Tang, Wei Xiang Jiang, Guo Dong Bai, Jian Tang, Lin Bai, Cheng‐Wei Qiu, Tie Jun Cui

**Affiliations:** ^1^ State Key Laboratory of Millimeter Waves School of Information Science and Engineering Southeast University Nanjing 210096 China; ^2^ Department of Electrical and Computer Engineering National University of Singapore Singapore 119620 Singapore

**Keywords:** far‐field radiation pattern, light‐controlled digital coding metasurfaces, real‐time control of electromagnetic waves

## Abstract

Since the advent of digital coding metamaterials, a new paradigm is unfolded to sample, compute and program electromagnetic waves in real time with one physical configuration. However, one inconvenient truth is that actively tunable building blocks such as diodes, varactors, and biased lines must be individually controlled by a computer‐assisted field programmable gate array and physically connected by electrical wires to the power suppliers. This issue becomes more formidable when more elements are needed for more advanced and multitasked metadevices and metasystems. Here, a remote‐mode metasurface is proposed and realized that is addressed and tuned by illuminating light. By tuning the intensity of light‐emitting diode light, a digital coding metasurface composed of such light‐addressable elements enables dynamically reconfigurable radiation beams in a control‐circuitry‐free way. Experimental demonstration is validated at microwave frequencies. The proposed dynamical remote‐tuning metasurface paves a way for constructing unprecedented digital metasurfaces in a noncontact remote fashion.

All the time, human beings explore to manipulate electromagnetic (EM) waves in a way they want. However, it is scarcely possible to achieve this goal by only using natural materials composed of atoms. Encouragingly, the emergence of metamaterial, an artificial material composed of periodically or nonperiodically arranged subwavelength elements, offers significant convenience for tailoring EM waves due to their unique EM properties that cannot be found in nature.^1^, ^2^, ^3^, ^4^, ^5^, ^6^ In the past decades, metamaterials described by macroscopic effective medium theory have attracted extensive attention and investigations, and have realized many intriguing physical phenomena such as negative refraction,^7^ backward wave propagation,^8^ perfect imaging,^9^, ^10^ optical illusion,^11^, ^12^, ^13^ and invisibility cloaking.^14^, ^15^, ^16^, ^17^, ^18^, ^19^ Though metamaterial has already demonstrated many novel physical properties and potential applications, the un‐negligible material loss, fabrication, and assembly challenges still exist for practical applications, especially at millimeter‐wave, terahertz, and even higher frequencies. Recently, a new class of two‐dimensional (2D) ultrathin metamaterials, termed as metasurfaces, has been developed with the flexible function of manipulating the wavefronts of EM waves.^20^, ^21^, ^22^, ^23^, ^24^ Especially, active metasurfaces allow a dynamical tuning of EM response.^25^, ^26^, ^27^, ^28^, ^29^ Metasurfaces can greatly reduce the material loss and overcome the fabrication issue, due to the fact that the thickness of a metasurface is usually only a fraction of the working wavelength. Despite being at the infancy, metasurfaces have shown many practical applications.^30^

Based on the effective medium theory, the medium parameters (e.g., permittivity and permeability) of existing metamaterials are continuous. By contrast, digital metamaterials which are described by digital “bits” and “bytes” have been proposed in 2014.^31^ In the meantime, the concept of using binary codes to quantify phase responses of metamaterial unit cells from 0 to 2π has been proposed and experimentally verified to construct coding metamaterial, digital metamaterial, and programmable metamaterial.^32^ To be noted, N‐bit metamaterials or metasurfaces need 2^N^ discrete phases to characterize the properties of digital units. For example, the unit cells of the simplest 1‐bit coding metasurfaces have two discrete phase states corresponding to two digits of “0” and “1,” which have 180° (or π) phase difference. By adopting appropriate coding mechanisms, namely, arranging the digital unit cells on 2D surface with different spatial or frequency coding sequences, different features of EM waves such as far‐field radiation, scattering, polarization state, and wave‐vector mode can be easily controlled.^33^, ^34^, ^35^, ^36^, ^37^, ^38^, ^39^, ^40^, ^41^, ^42^

To achieve digital unit particles with state‐switchable properties, tunable active devices have been embedded in the digital coding units, such as diodes,^25^, ^43^ electromagnets,^44^ and microelectromechanical system.^45^, ^46^ We remark that most of reported controlling mechanisms for tunable digital units need an external direct current (DC) supply device and a series of connecting wires, which may increase the complexity of the whole system and bring in problems to the control and movement of the digital coding metamaterials or metasurfaces. In addition, it is very challenging to realize remote controls of multifunctional digital metamaterials or metasurfaces, due to the fact that there are many wires connected between the DC supply and the interfaces embedded in the digital metamaterials or metasurfaces. In view of this, to realize reconfigurable digital metamaterials or metasurfaces which can be controlled remotely, an optical‐control method is highly desirable. Actually, some attempts have been made to remotely manipulate the functionalities of metamaterials and devices in microwave frequency and direct current through illuminating light.^47^, ^48^, ^49^

Here, we propose and design a kind of light‐controllable digital coding metasurfaces. First, we present and numerically design a light‐tunable digital unit particle. By controlling the intensity of the illumination light, the reflection phase of the proposed digital particle can be dynamically tuned as desired. As a proof of the concept, a light‐dependent reflective digital metasurface is fabricated and measured. The proposed light‐controllable digital metasurface serves as an alternative approach to control the EM waves in real time, and opens novel possibilities to realize remotely tunable digital coding metasurfaces.

The proposed light‐controlled metasurface is composed of an array of tunable digital unit particles that are arranged in the *x* and *y* directions, together with an array of photodiodes which provides light‐controllable DC bias for the varactor diodes embedded in the digital particles, as shown in **Figure** **1**a. We first predesign the coding sequence and send the signal by light source array, then the photodiode array receive the coding sequence and transmit it to the metasurface, and finally the incident waves will be dynamically controlled by the digital coding metasurface in the microwave region. Hence, the scattered or radiated waves are controlled by the “coded” light emitted by the source array.

**Figure 1:**
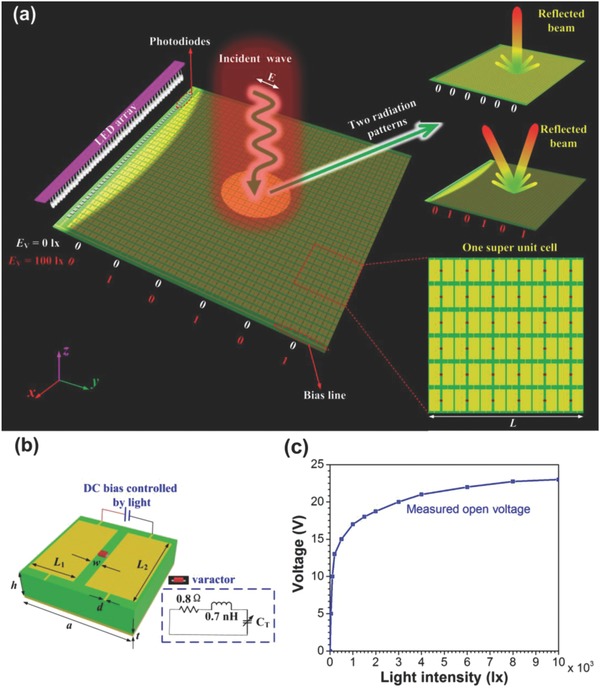
a) Illustration of the proposed light‐controlled digital coding metasurface. By remotely changing the intensity of the illumination light, the voltage generated by the photodiodes can be dynamically controlled. Upper‐right inset: when the light intensity *E*_V_ = 0 lx, the reflected beams of the light‐controlled digital metasurface with coding sequence of “000000.” Middle‐right inset: when the light intensity *E*_V_ = 100 lx, the reflected beams of the light‐controlled digital metasurface with coding sequence of “010101.” Lower‐right inset: a super unit cell which is composed of 6 × 6 digital particles. b) Unit cell of the light‐controllable digital metasurface. The DC bias of the varactor is provided by the photodiodes which are controlled by LED array. The inset shows equivalent lumped elements model of the varactor. c) The measured relationship between the bias voltage generated by photodiode array and the intensity *E*_V_ of illumination light.

The idea of using the “coding” unit cell to control EM waves was proposed in ref. 32. Assuming that the digital metasurface is composed of *N* × *N* super units, when the plane wave is incident normally on the metasurface, the expression of far‐field radiation pattern *F*(θ, φ) is written as

(1) $$F\left(\theta ,\phi \right)=\sum_{m=1}^{N}\sum_{n=1}^{N}Ae^{-i\left[\left(\frac{2\pi }{\lambda }L\left(m-1/2\right)\sin\theta \cos\phi +\frac{2\pi }{\lambda }L\left(n-1/2\right)\sin\theta \sin\phi \right)+\varphi \left(m,n\right)\right]}$$

in which *A* is the reflection amplitude of the super unit and its value is 1; ϕ(*m*, *n*) is the reflection phase of the super unit. For the “0” element, ϕ(*m*, *n*) = 0; for the “1” element, ϕ(*m*, *n*) = π. *L* is the side length of the super unit, and λ is the working wavelength.

If the number of the units is finite, we can simplify the far‐field radiation pattern |*F*(θ, φ)|. The function |*F*(θ, φ)| will achieve the maximum value, when and only when the elevation angle θ and azimuth angle φ satisfy the following equations,

(2) $$\phi \;\;=\;\;\pm \arctan\frac{\Gamma_{x}}{\Gamma_{y}},\;\;\phi =\pi \pm \arctan\frac{\Gamma_{x}}{\Gamma_{y}}$$

(3) $$\theta \;\;=\;\;\arcsin\left(\lambda \sqrt{\frac{1}{\Gamma_{x}{}^{2}}+\frac{1}{\Gamma_{y}{}^{2}}}\right)$$

where Γ_*x*_ and Γ_*y*_ are physical periodic lengths of the phase gradient coding sequence along the *x* and *y* directions, respectively.

To realize the above‐mentioned light‐controlled digital metasurface, we propose and design a digital particle which is illustrated in Figure 1b, composing of two symmetrical metallic patches on the top and the metallic ground on the bottom. Each metal patch has two stubs at its ends, serving as bias lines. For realizing tunable characteristics, a varactor diode is loaded in the gap to connect the two symmetrical patches. To tune the capacitance (*C*_T_) of the embedded varactor with light, we use an array of silicon positive‐intrinsic‐negative (PIN) photodiodes operating in photovoltaic mode to provide DC reverse voltage (*V*_R_).

The designed light‐tunable digital particle is simulated and optimized in the software CST Microwave Studio. The substrate of the digital particle has the dielectric constant of 2.65 with the loss tangent of 0.001, and the thickness *h* is chosen as 3.0 mm. The side length of the digital particle is *a* = 12.0 mm and the thickness of symmetrical metallic patches and ground is *t* = 0.018 mm. The width of the bias line is chosen as *d* = 0.2 mm, and the gap between the patches is *w* = 1.2 mm. In the simulations, because there is no existing model to mimic the varator diode in commercial package, we use lumped element model: a resistor‐inductor‐capacitor (RLC) series circuit, as shown in the inset of Figure 1b, to simulate the varactor diode. Some parasitic parameters can be found from the handbook,^50^ parasitic resistance Rs = 0.8 Ω, parasitic inductance Ls = 0.7 nH. The variable capacitance *C*_T_ is dependent on the bias voltage. To realize the desired binary elements, we make a good deal of numerical simulations based on different value of *L*_1_, *L*_2_, *C*_T_, and choose *L*_1_ = 5.1 mm, *L*_2_ = 10.0 mm, and two optimal values of *C*_T_, 2.67 and 0.95 pF, by observing the reflection phase of the designed digital particles. For *C*_T_ = 2.67 pF and *C*_T_ = 0.95 pF, the simulated amplitudes and phases of the reflection coefficient (*S*_11_) of the designed digital particles are shown in **Figure** **2**. It is observed that the phase difference between the *C*_T_ = 2.67 pF curve and the *C*_T_ = 0.95 pF curve ranges from 160° to 196° from 3.69 to 4.10 GHz (about 10.2% relative bandwidth) and approaches 180° at 3.75 and 4.02 GHz. In addition, at these two frequency points, the reflection amplitudes are larger than −1.22 dB and the largest reflection amplitude is −0.23 dB. We encode the digital particle with *C*_T_ = 2.67 pF as “0” unit and that with *C*_T_ = 0.95 pF as “1” unit.

**Figure 2:**
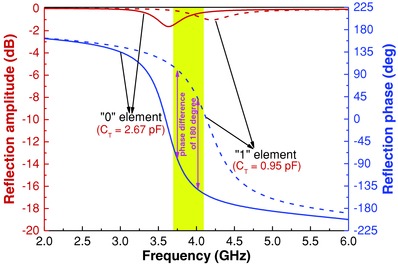
Simulated reflection amplitudes and phases of the designed “0” and “1” element from 2.0 to 6.0 GHz, respectively. The yellow area presents the phase differences of the designed “0” and “1” element, ranging from 160° to 196°, when the frequency changes from 3.69 to 4.10 GHz. The two violet double arrow lines indicate the phase difference of the designed “0” element and “1” element. Obviously, the phase difference is ≈180° at 3.75 and 4.02 GHz.

To satisfy the dynamic capacitance requested by the coding particle, we choose to use the varactor from Skyworks SMV1405‐079LF, in which the capacitance can be tuned from 0.63 to 2.67 pF. The maximum reverse bias voltage of this varactor diode is 30 V. The photodiode, BPW‐34‐S, is used to provide the needed bias voltage. Each photodiode generates the voltage of 0.5 V, so we connect 50 photodiodes in series to provide the requested voltage. To excite the photodiodes, we use an array of low‐power light‐emitting diodes (LEDs) as the light source. By changing the intensity of light emitted by LEDs, the voltage generated by the photodiodes can be dynamically controlled.

To investigate the relationship between the voltage and the light intensity, we test the generated voltage in experiment when the intensity of the light changes, and the measured results are shown in Figure 1c. It is observed that when the illumination intensity (*E*_V_) of LEDs increases gradually from 0 to 10^4^ lx, the generated voltage changes accordingly from 0 to 23.0 V. In this range, from the handbook, we know the capacitance of the selected varactor can be tuned from 2.67 to 0.77 pF.

Next, we make numerical simulations to verify the beam‐modulating performance of the designed light‐dependent digital metasurface, which can be divided into 6 × 6 super unit cells. Each super unit cell is composed of 6 × 6 subparticles, as shown in the inset of Figure 1a. Each subparticle has two states “0” and “1.” In one super unit cell, the states of the subparticles are the same. Hence, the states of the super unit cell can be described by “0” and “1.” In our simulations, the subparticle with a capacitor *C*_T_ = 2.67 pF denotes “0” unit, and the sub‐particle with a capacitor *C*_T_ = 0.95 pF denotes “1” unit.

According to Equation (1), for the coding sequence “000000,” the digital metasurface has no phase changes along the *x* and *y* directions, i.e., Γ_*x*_ → ∞ and Γ_*y*_ → ∞, and we can get θ = 0°. Hence, a main reflection beam will be generated by the digital metasurface. For 1D coding sequence “010101” along the *y* direction, i.e. Γ_*x*_ → ∞ and Γ_*y*_ = 2*L*, we obtain φ = 90° and φ = 270°. In our design, the side length is *L =* 72 mm, about one wavelength at 3.75 and 4.02 GHz, and hence we obtain θ = 30°. In this case, two main reflection beams will be generated at (30°, 90°) and (30°, 270°), respectively.

The simulation results of the digital coding metasurfaces defined by the coding sequences “000000” and “010101” are shown in **Figure** **3**. At the working frequency 3.75 GHz, for the coding sequence “000000,” we observe that the main beam is directed to the *z*‐axis, as shown in Figure 3a; for the coding sequence of “010101,” two main beams on the *yoz*‐plane are observed in Figure 3b. The full‐wave simulation results are the same as those predicted by the above theory. We also observe the numerical results at 4.02 GHz for the coding sequences “000000” and “010101,” as shown in Figure 3c,d. Hence, the presented digital coding metasurface can work in a frequency band instead of only one frequency point. Our propsed light‐controlled digital metasurface can also realize other functions, and more complicated radiation patterns of the digital coding metasurface are shown in Figure S1 of the Supporting Information. More recently, the digital coding metasurfaces were used to realize real‐time holographic imaging.^41^ In our work, the light‐controlled digital metasurfaces can also be used to generate the holographic images. In ideal case, the phase response of each super unit cell in the digital coding metasurface can be controlled independently, and hence different binary phase distribution patterns are generated for different codings. Therefore, combining with the corresponding phase recovery algorithm (such as GS algorithm), the light‐controlled coding metasurfaces can produce some corresponding hologram. Under far‐field radiation of the horn antenna, a holographic image can be reproduced corresponding to the original image.

**Figure 3:**
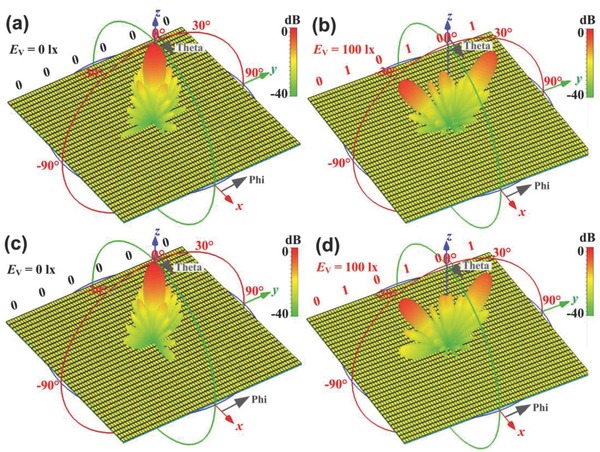
Simulated reflection beams of the light‐controlled digital coding metasurface at 3.75 GHz with a) coding sequence of “000000,” b) coding sequence of “010101,” and 4.02 GHz with c) coding sequence of “000000,” d) coding sequence of “010101.”

To experimentally verify the proposed digital coding metasurface controlled by remote illuminating light, we have fabricated the LED‐based light source, the photodiode array, and the metasurface. As mentioned previously, we need 50 photodiodes connected in series to provide enough bias voltage to one variode. We assume that the subparticles in each super unit cell have the same state. Then each super unit cell can share the bias voltage. To control the 6 × 6 super unit cells independently, we need 36 × 50 photodiodes to provide the bias voltage, which is too complicated. Details of scheme for controlling 6 × 6 super unit cells of the digital coding metasurface individually by remotely illuminating light are shown in Figure S2 of the Supporting Information. To provide bias to the varactor diode, there are reserved stubs at the upper and lower edges of each patch, respectively. Hence, the upper and lower patches are connected by the stub. In such a case, each row of digital particles can be controlled by two feeding lines and these feeding lines for every row are connected together. To simplify the fabrication, as a proof of concept, we use only one string of photodiodes as the voltage source. There are six columns in the fabricated digital metasurface along the *y* direction, and each column contains six super unit cells along the *x* direction, which share the same bias voltage. To realize the coding sequences “000000” and “010101,” we connect the 2nd, 4th, and 6th columns to the string of photodiodes. Hence, when the intensity of the light is 0 lx, the bias voltage is 0 V, and the metasurface will act as sequence “000000”; when the intensity of the light is 100 lx, the bias voltage is 10 V, and the metasurface will act as sequence “010101.” The fabricated sample totally covers an area of 452 × 452 mm^2^ (6.06 × 6.06 λ^2^ at the operating frequency of 4.02 GHz).

We fabricate the digital metasurface, and there are two 2.0 mm wide bias lines connected to the positive and negative electrodes of the photodiode array, which is located by the side of metasurface, as shown in **Figure** **4**a. To illuminate the photodiode array, we also fabricate an array of 50 low‐power white LEDs as a tunable handcrafted light source, which is shown in Figure 4b.

**Figure 4:**
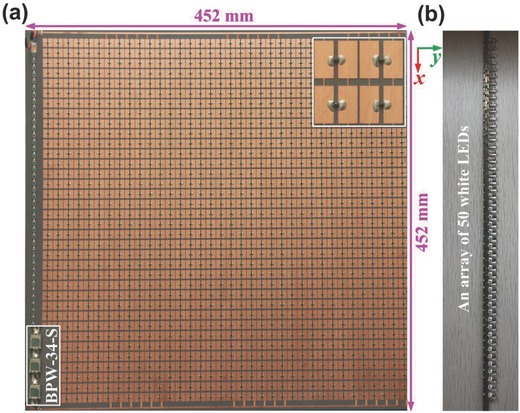
Photographs of the fabricated sample and the handcrafted light source. a) Fabricated sample consists of the light‐controlled digital metasurface, photodiodes, and two bias lines, which covers an area of 452 × 452 mm^2^. The upper right and lower left insets show some details of digital particles and BPW‐34‐S photodiodes, respectively. b) Fabricated tunable handcrafted light source comprised of an array of 50 white LEDs.

We measure the far‐field radiation beams of the fabricated digital coding metasurface in the microwave chamber, and the photograph of the experimental setup is shown in **Figure** **5**a. A horn antenna with gain of 15 dBi, working from 3.22 to 4.90 GHz, is used as the exciting source, which is placed 2.4 m (about 32 wavelengths) away from the metasurface. In this case, the incident wave on the light‐controlled digital metasurface can be regarded as a quasi‐plane wave. First, when the light intensity is 0 lx, the bias voltage is 0 V, and the measurement result of the coding metasurface with sequence “000000” is shown in Figure 5b. In such a case, the normally incident plane waves is directly reflected back, and the half power (3 dB) beam width (HPBW) of the reflected beam is ≈10.2° at 3.75 GHz. Then, we turn the light intensity to 100 lx, and the bias voltage is 10 V. In such a case, the metasurface is coded with sequence “010101,” and it is observed that there are two main reflected beams (around ±30°) with HPBW being approximately 8.4° in the measured far‐field radiation pattern at 3.75 GHz, as shown in Figure 5d.

**Figure 5:**
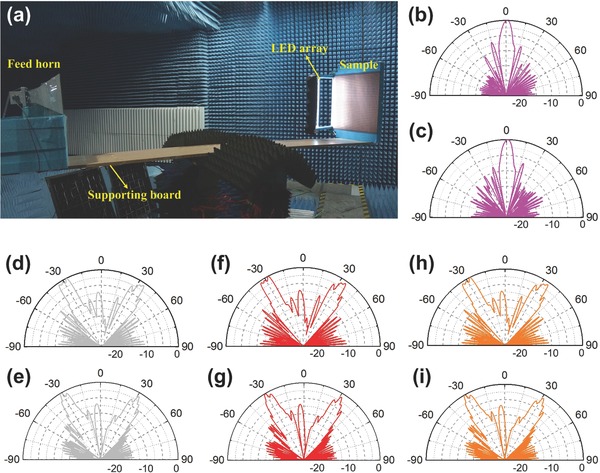
Experimental setup and measurement results of the light‐controlled digital coding metasurface. a) The experimental setup. When light source turns off, the measured reflection beams of the light‐controlled digital metasurface. b) 3.75 GHz, c) 4.02 GHz. Measured reflection beams of the light‐controlled digital metasurface at 3.75 GHz as illuminating the metasurface with d) white light, f) red light, h) yellow light. Measured reflected beams of the light‐controlled digital metasurface at 4.02 GHz as illuminating the metasurface with e) white light, g) red light, and i) yellow light.

We also measure the far‐field radiation patterns of the light‐controlled digital metasurface at other frequencies. The measured results at 4.02 GHz for the coding sequences “000000” and “010101” are shown in Figure 5c,e. It is verified again that the digital metasurface can realize different reflection beams by controlling the light source.

Furthermore, we also fabricate two other low‐power LED sources to emit red and yellow lights (monochrome light at different optical frequencies). We measure the far‐field radiation patterns of the light‐controlled digital metasurface when the red light and yellow light are illuminating on the photodiodes, respectively. The results are shown in Figure 5f–i, from which we observe that all measured far‐field radiation patterns of the digital coding metasurface have two main reflected beams. All these measured reflection beams of the light‐dependent digital coding metasurface are in good agreements with the simulation results, which validates the feasibility of the light‐controlled digital coding metasurface.

In summary, we proposed and verified the concept of optically remote control of far‐field radiations. A designed digital particle structure was proposed, which has ≈180° reflection phase difference when the intensity of illumination light is changed. Adopting such digital particles as composing units, a light‐dependent reflection‐type digital metasurface has been fabricated and measured. Good agreement between measurement and simulation results demonstrates that the scattered or radiated property of the digital metasurface can be remotely reconfigured as the state of light source is switched between different states. It should be noted that, in the current experiment, the maximum reverse bias voltage of the chosen varactor diode is 30 V and each photodiode generates the voltage of 0.5 V. Hence we connect 50 photodiodes in series to provide the requested voltage. For convenience and low cost, we choose the varactor diode with model “Skyworks SMV1405‐079LF” and photodiode “BPW‐34‐S.” If we choose other diodes that request low bias voltage and advanced photodiodes that can generate higher voltage, it is possible to simplify the complicated biasing wire network or make the circuitry free. In an ideal case, one photodiode can generate enough voltage requested by the diode, and we can integrate the photodiode and diode into the digital particle. Hence, the proposed scheme is possible to overcome the complicated biasing wire network used to control each individual particle in the digital metasurface.

## Experimental Section

In measurement, the transmitting horn antenna and fabricated digital metasurface are fastened on a supporting board, which has similar EM property to the air. The supporting board is fixed on a mechanical turntable, which is controlled by a computer and can be rotated by 360° in the horizontal plane with high precision. When the supporting board carrying the transmitting antenna and the sample rotates from −90° to 90°, the receiving antenna which is mounted on a tripod in the far‐field region receives the electric fields in the horizontal plane (*yoz*‐plane) with the angular resolution of 0.1°. In addition, when the handcrafted low‐power LED arrays is used to illuminate the photodiode array, the LED array is fixed 20 mm right above the plane of the photodiode array, and the light intensity of the LED array is gradually increased until the DC reverse voltage generated by the photodiodes goes up to 10 V. When the voltage reaches 10 V, the total power of the 50 low‐power LEDs is ≈750 mW, which indicates that the light‐controlled digital coding metasurface is energy efficient.

## Conflict of Interest

The authors declare no conflict of interest.

## Supporting information

Supplementary
