# Supplementary material for: Light‐Controllable Digital Coding Metasurfaces
Source: Adv Sci (Weinh). 2018 Aug 1;5(11):1801028. doi: 10.1002/advs.201801028 (PMC6247069; doi:10.1002/advs.201801028)
Supplement: Supplementary file 1 — Supplementary [file ADVS-5-1801028-s001.pdf]

Copyright WILEY-VCH Verlag GmbH & Co. KGaA, 69469 Weinheim, Germany, 2018.

Supporting Information

for *Adv. Sci.,* DOI: 10.1002/advs.201801028

Light-Controllable Digital Coding Metasurfaces

Xin Ge Zhang, Wen Xuan Tang, Wei Xiang Jiang, Guo Dong Bai, Jian Tang, Lin Bai, Cheng-Wei Qiu,* and Tie Jun Cui**

Supporting Information

Light-controllable Digital Coding Metasurfaces

Xin Ge Zhang, Wen Xuan Tang, Wei Xiang Jiang, Guo Dong Bai, Jian Tang, Lin Bai, Cheng-Wei Qiu* and Tie Jun Cui**

In this Supporting Information, we provide some details of other functions of the proposed digital metasurface controlled by remote-mode illuminating light and we also discuss the bias wire network of such an advanced digital metasurface.

The proposed light-controlled digital coding metasurface can generate more radiation patterns, due to the 6×6 super unit cells can be controlled independently. It can be verfied in numerical simulations, because we can arrange the "0" and "1" super unit cells on 2D surface with different spatial coding sequences. We present some simulated radiation patterns that corresponding to different coding sequences of the digital coding metasurface, as shown in **Figure S1**. It is observe that the proposed digital coding metasurface can indeed realize more complicated patterns as the coding sequences of the metasurface changes.

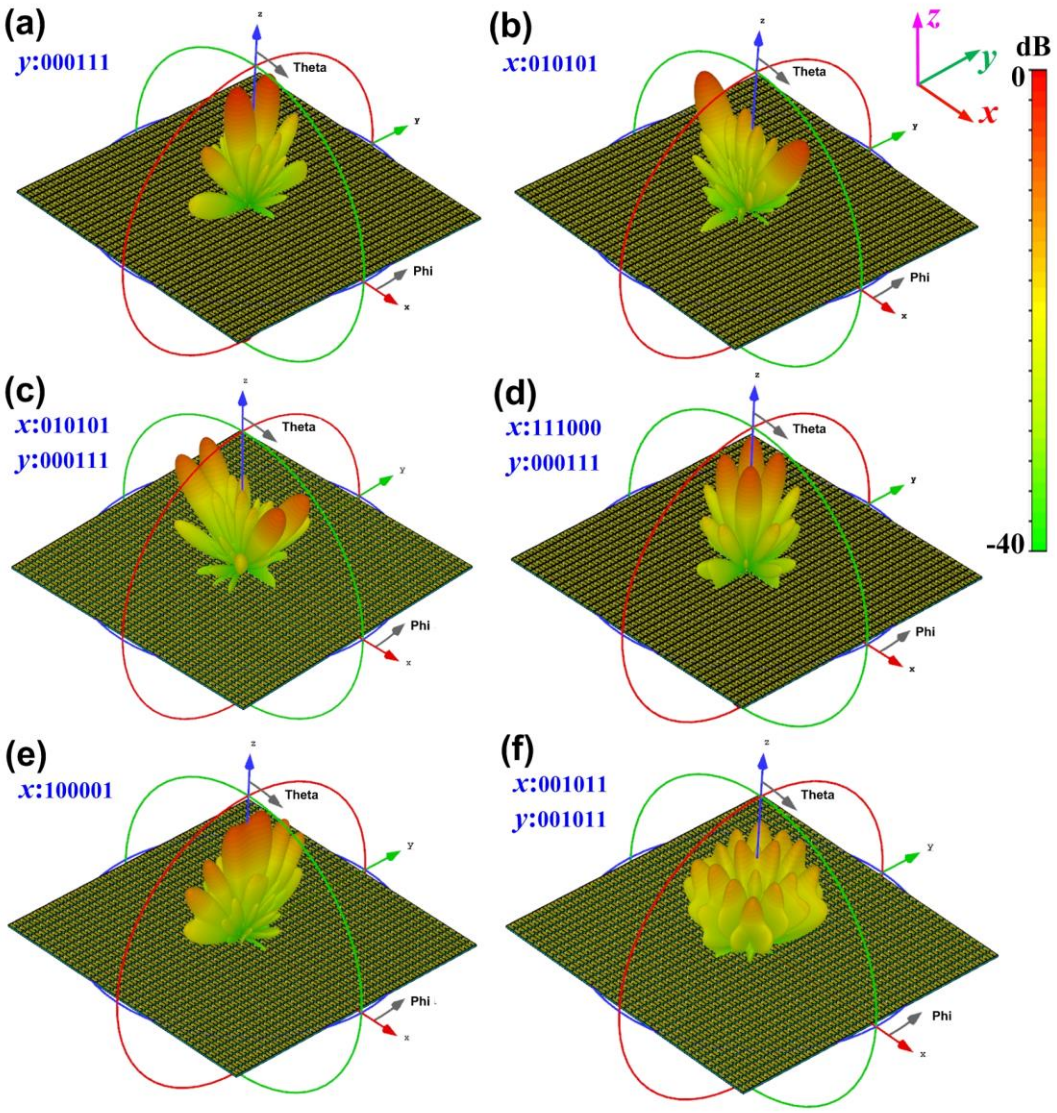

Figure S1. Simulated radiation patterns of the light-controlled digital coding metasurface at 3.75 GHz with (a) coding sequence of "*y*: 000111", (b) coding sequence of "*x*: 010101", (c) coding sequence of "*x*: 010101, *y*: 000111", (d) coding sequence of "*x*: 111000, *y*: 000111", (e) coding sequence of "*x*: 100001", (f) coding sequence of "*x*: 001011, *y*: 001011".

To experimentally realize the multifunctional light-controlled digital coding metasurface that can generate more radiation patterns, comparing with the current sample presented in main text, we need to redesign the DC bias network and using 36 photodiode arrays and LED arrays to provide 36-channel DC voltages. We have designed the new scheme in details for controlling 6×6 super unit cells of the digital metasurface individually by remote illuminating light, as shown in **Figure S2**.

To observe the connection of the DC bias lines for the super unit cells of the digital metasurface more clearly, we present the amplifying spacing among each super unit cells and the detail of a super unit cell, as shown in **Figures S2b and S2c.** The actual structure of one super unit cell and the connection mode of its internal bias lines are shown in **Figure S2c**. From **Figure S2**, we observe that the 6 top stubs of the first super unit cell in the upper left corner is connected to a bias line (red line) that is connected to the positive electrode of one photodiode string, and the other 35 super unit cell are also connected to the positive electrode of the remaining 35 photodiode strings in the same connection mode, respectively. Additionally, 6 super unit cells of each row along the *y* direction are connected to one same grounded line (white line) through their respective 6 bottom stubs. Therefore, there are 6 grounded lines of the digital coding metasurface, and all these grounded lines are connected to the same grounded line (see **Figure S2a**), and then connected to the negative electrodes of the 36 photodiode strings. To drive the 36 photodiode strings independently, we need to fabricate 36 LED strings for illuminating the 36 photodiode strings, respectively. By using this bias network and 36×50 photodiode and LED pairs, the 6×6 super unit cells of the digital coding metasurface can be independently tuned as “0” element or “1”element.

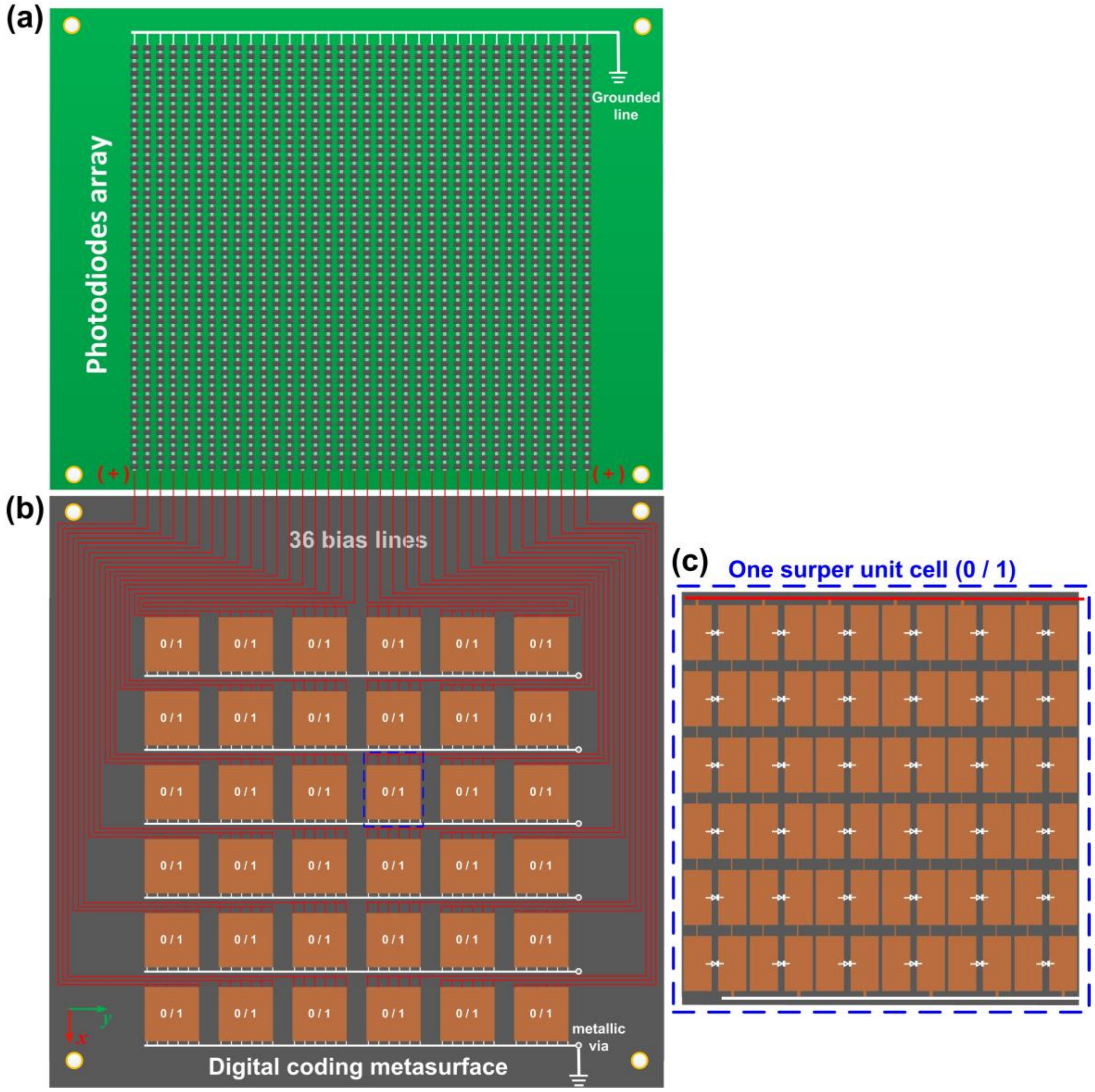

Figure S2. (a) 36 rows of LED strings are used to provide 36-channel DC voltages. (b) The scheme of the multifunctional digital coding metasurface in which the 6×6 super unit cells can be controlled independently by remote illuminating light. (c) The structure of one super unit cell and the connection mode of its internal bias lines.
